# Doctors’ satisfaction with the rehabilitation system for anti-terrorist operation participants: A factor analysis

**DOI:** 10.1016/j.heliyon.2024.e40667

**Published:** 2024-11-23

**Authors:** Irina Holovanova, Oleksandr Havlovsky, Shanshan Wang, Oleksandr Korneta, Maksym Khorosh, Igor Kaydashev, Renee Robinson, Ubydul Haque

**Affiliations:** a Poltava State Medical University, Poltava, Ukraine; b Department of Population & Community Health, College of Public Health, University of North Texas Health Science Center, Fort Worth, TX, USA; c University of Alaska Anchorage, College of Pharmacy, 2533 Providence Drive, PSB 108B, Anchorage, AK, 99508, USA; d Rutgers Global Health Institute, Rutgers School of Public Health, New Brunswick, NJ, USA; e Department of Biostatistics and Epidemiology, School of Public Health, Rutgers University, Piscataway, NJ, USA

**Keywords:** Rehabilitation, Anti-terrorist operation (ATO), Veterans

## Abstract

**Background:**

A war has a catastrophic effect on veterans’ health. However, there is a knowledge gap about the veterans’ rehabilitation system, requirements, and the satisfaction of doctor-organizers. The present study aims to assess the level of satisfaction of the doctor-organizers of health care with the existing rehabilitation system for anti-terrorist operation (ATO) veterans in Ukraine.

**Methods:**

A cross-sectional design was conducted during the ATO in March–April 2020. A total of 25 organizing doctors from healthcare institutions filled out the form. The questionnaire consisted of three blocks: 1) Main characteristics of the existing system of rehabilitation of ATO veterans (14 items); 2) Strengths of the existing system of rehabilitation of ATO veterans (11 items); and 3) Weaknesses of the existing system of rehabilitation of ATO veterans (8 items). Evaluation of the rehabilitation system was carried out using factor analysis.

**Results:**

The main features of the system were accessibility, financing of rehabilitation programs, and commitment to the rehabilitation of ATO veterans. The advantages of the system included 1) doctors and organizers identifying the regulatory and legal provision of medical and social protection for the rehabilitation of veterans, 2) access to experienced personnel to carry it out, 3) respectful attitude towards veterans, and 4) comprehensive rehabilitation. In addition, disadvantages inherent in the system included: 1) violation of the integral principle, 2) insufficient material, technical support, and financial resources for rehabilitation programs, and 3) legislative and regulatory gaps necessary to provide rehabilitation to ATO veterans.

**Conclusions:**

The findings provided healthcare organizers with information on the main features, advantages, and disadvantages of the rehabilitation of ATO veterans. These insights can aid in optimizing the current rehabilitation system in Ukraine.

## 1 Introduction

Armed conflicts have a catastrophic effect on the health and well-being of nations, including Ukraine [[1], [2], [3], [4], [5], [6], [7]]. The Russian Federation began attacks against Ukraine in February 2014, which led to an increase in the number of military and civilian injuries, and the need for rehabilitation services and supports [8]. Every year on the battlefields in Ukraine, 0.3–3.4 % of over 1 million soldiers received spine injuries [9]. 16–18 % of soldiers sustained limb injuries with severe damage to peripheral nerves [9]. About 25 % of soldiers suffered contusions of the brain and spinal cord [9]. Additionally, 15 % of soldiers experienced damage to the radial nerve and 32–68 % of soldiers endured complex injuries [9]. In 2020–2021, Ukrainian doctors diagnosed 72 % of war veterans with asthenic-vegetative syndromes, and 46 % were diagnosed with cognitive disorders [10].

According to the experience of developed countries, the treatment of victims during armed conflicts has led to many medical advances [[11], [12], [13]]. These advances have benefited both the civilian health care system (CHS) and the military health care system (MHS) [[11], [12], [13]]. The regime of anti-terrorist operations (ATO) was introduced in the eastern regions of Ukraine in 2014 [14]. At the beginning of the ATO, the healthcare system of Ukraine needed rapid and radical optimization, as wounded and injured servicemen needed medical assistance. From 2014 to 2022, in the area where the ATO was carried out, armed confrontation continued, from which servicemen suffered the most. This led to an increased need for medical assistance and rehabilitation services [15]. The government of the country, responding to the requirements of the time, created and continued to improve the legal framework for providing medical care to this population [16]. Meanwhile, doctor-organizers who established rehabilitation services worked with military leaders to implement the legal framework into daily practice, addressing gaps identified by providers in the rehabilitation system of ATO participants [17].

Respectful treatment of the veteran by the health care service expresses that it values what they have experienced, and is sympathetic to their experience [18]. The WHO evaluation mission on rehabilitation, which was conducted in Ukraine in December 2015, concluded that the country lacks a comprehensive rehabilitation system [17,19]. Additionally, legislation on rehabilitation in Ukraine is fragmented and lacks coordination between authorized ministries and organizations [17,19]. It is important to note that the existing rehabilitation system did not meet the requirements of veterans, necessitating changes to the system. The doctors’ experiences were crucial in making decisions about the reorganization and reforms in the rehabilitation system for war veterans. Thus, the current study aims to evaluate doctor-organizers’ satisfaction with the existing rehabilitation system for veterans in Ukraine, focusing on its main features, advantages, and disadvantages.

## 2 Methods

**Fig. 1:**
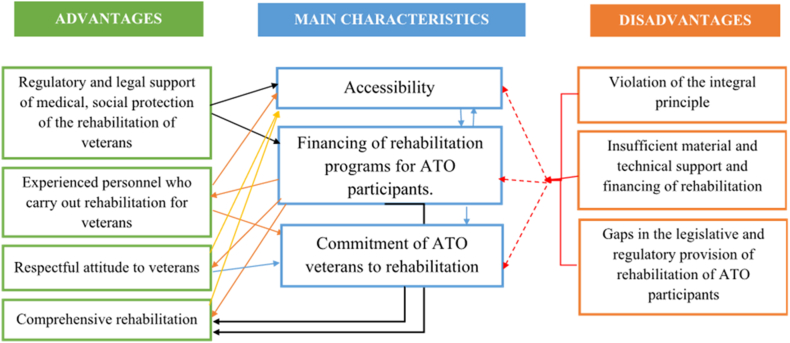
The conceptual model of rehabilitation system for ATO participants according to results of focus group and the factor analysis. Abbreviations: ATO: anti-terrorist operation.

A cross-sectional, quantitative study was conducted in March–April 2020. A focus group of 25 doctors who treated both war veterans and civilians was conducted to create a questionnaire assessing providers’ opinions on the rehabilitation system [17]. The doctors selected for this study work in hospitals closely involved in rehabilitating war veterans and disabled persons, including the Poltava Regional Clinical Hospital, the Kremenchuk Regional Hospital, the Lubny Regional Hospital for War Veterans, and the Hospital of the Ministry of Internal Affairs. The conceptual model of the rehabilitation system for ATO participants according to the results of the focus group and the factor analysis is shown in Fig. 1. A total of 25 doctor-organizers of health care institutions filled out the questionnaire, including six directors of veteran hospitals and their deputies from Poltava, Kremenchuk, and Lubniv (Central region of Ukraine); three doctors from the Hospital of the Ministry of Internal Affairs who are in direct contact with veterans; seven deputy directors of central district hospitals; and nine general practitioners who provide medical assistance to former military personnel. Ethical approval was obtained from Poltava State Medical University, Ukraine. Written informed consent was obtained from each participant.

The questionnaire consists of three blocks: 1) Main characteristics of the existing system of rehabilitation of ATO veterans; 2) Strengths of the existing system of rehabilitation of ATO veterans; and 3) Weaknesses of the existing system of rehabilitation of ATO veterans. The first block considers the characteristics that distinguish the rehabilitation of veterans from that of civilians (14 items). After completing the first block, the respondent proceeded to the second block (11 items) and finally to the third block (8 items). Items were scored on a 5-point Likert scale with the following response options: 0 = strongly disagree, 1 = disagree, 2 = neutral, 3 = agree, and 4 = strongly agree.

Data entry and statistical analysis were performed using IBM SPSS version 25.0. The process of extracting factors from the correlation matrix using principal component analysis led to the identification of factors. Factor analysis is a collection of statistical techniques to identify the number of distinct constructs required to explain the pattern of correlations among a set of measures. Mathematically, it examines equations that maximize the multiple correlations between factors and items. Factor analysis can be viewed as an extension of principal component analysis, as both methods approximate the covariance matrix among variables. A correlation matrix shows the relations between multiple variables, indicating how changes in one variable are linked to changes in another. We used Spearman’s rank correlation coefficient (r) to determine the main features of the rehabilitation of ATO participants. A probability of <0.05 was considered statistically significant.

## 3 Results

The demographic and professional characteristics of the respondents are presented in Table 1. The sample included 25 doctors, with 76 % women, a mean age of 49.6 years, a mean medical experience of 23.8 years, and the meaningful experience of medical care for ATO veterans of 5.1 years (Table 1).

**Table 1:** Demographic and professional characteristics of respondents (heads of healthcare institutions and doctors).

Characteristic	Values
Women, n (%)	19 (76 %)
Men, n (%)	6 (24 %)
Length of medical experience (years), mean (95 % CI)	23.8 (18.3–29.2)
The experience of medical care for ATO veterans (years), mean (95 % CI)	5.1 (4.5–5.8)
Middle age (years), mean (95 % CI)	49. 7 (44.0–55.4)

A correlation matrix was created to determine the main features of the rehabilitation of ATO participants (Table 2). ‘Allocation of funds for the implementation of the program’ correlates with ‘Creation of material and technical conditions for providing assistance to ATO veterans’ (r = 0.795), which shows a high dependence of the provision of rehabilitation assistance on the availability of a sufficient level of funding. ‘Priority and availability of medical care for ATO participants’ is considered as an important prerequisite for the ‘Development of a physical and mental health rehabilitation program’, and the two items are correlated (r = 0.627), indicating that, with the presence of an emphasis on providing assistance to ATO participants in the paradigm of providing medical care, there would be a strengthening of existing and development of new adequate approaches to rehabilitation care. ‘Development of a physical and mental health rehabilitation program’, in turn, correlated with ‘Accessibility to sanatorium-resort treatment’ (r = 0.727) and ‘Accessibility to a discounted purchase of medicines’ (r = 0.726). This suggests that as funding for rehabilitation programs is strengthened and expanded, treatment becomes more accessible (with an increased availability of preferential spots for veterans), and the network of pharmacies participating in programs aimed at preferential medicine provision for ATO veterans expands (providing partial or full coverage of medication costs with a prescription from a specialized physician). ‘Availability of sanatorium-resort treatment’ correlates with ‘Availability of places for the preferential purchase of medicines’ (r = 0.764), ‘Referral for treatment to the Medical Rehabilitation Center’ (r = 0.669), and ‘Independent application for medical help’ (r = 0.624). It is important to note that the availability of spa treatment (rehabilitative wellness therapy) serves as a key motivating factor for seeking rehabilitation care, as the opportunity to receive spa treatment increases the interest of individuals in pursuing assistance, and as a result, a greater overall treatment effect. Considering concomitant diseases common among veterans, they receive medical care from ‘Highly qualified medical specialists’, which correlates with both ‘Provision of highly specialized care to ATO veterans’ (r = 0.672) and ‘Referral for treatment to a medical rehabilitation center’ (MRC) (r = 0.720). The presence of highly qualified specialists in rehabilitation care facilities increases victims’ willingness to receive this type of care, as it strengthens their faith in a positive outcome and gives them hope for successful rehabilitation.

**Table 2:** Interrelationships of the main characteristics of the existing system of rehabilitation of ATO participants.

Item number	The main characteristics of the existing system of rehabilitation for participants of ATO	1	2	3	4	5	6	7	8	9	10	11	12	13	14
1	Allocation of funds for the implementation of the program	1.000													
2	Priority and availability of medical care for ATO participants	0.017	1.000												
3	Development of a physical and mental health rehabilitation program	−0.168	**0.627**	1.000											
4	Creation of material and technical conditions for providing assistance to ATO veterans	**0.795**	−0.010	−0.048	1.000										
5	Availability of sanatorium-resort treatment	0.145	0.511	**0.727**	0.029	1.000									
6	Availability of discounted drugs	0.247	**0.640**	**0.726**	0.199	**0.764**	1.000								
7	Availability of a psychologist at the place of service	−0.248	0.118	0.310	−0.389	0.550	0.197	1.000							
8	Providing highly specialized care to ATO veterans	0.405	−0.232	−0.020	0.341	0.064	−0.100	0.218	1.000						
9	Referral for treatment at the Medical Rehabilitation Center	0.407	0.417	0.472	0.411	**0.669**	0.410	0.167	0.443	1.000					
10	Free medical care	0.461	−0.017	0.320	0.588	0.378	0.284	−0.110	0.418	**0.696**	1.000				
11	Comfortable conditions for ATO veterans	0.506	0.204	0.097	0.431	0.339	0.256	0.049	0.489	**0.668**	0.18	1.000			
12	Highly qualified medical specialists	0.431	0.191	0.290	0.367	0.355	0.126	0.042	**0.672**	**0.720**	0.422	0.640	1.000		
13	Independent application for medical assistance	−0.002	0.583	0.521	0.152	**0.624**	0.399	0.077	0.049	**0.744**	0.36	0.448	0.382	1.000	
14	Following the doctor’s recommendations	0.371	0.310	−0.006	0.445	0.104	0.284	−0.269	0.247	0.365	0.203	0.538	0.286	0.494	1.000

Abbreviations: ATO: anti-terrorist operation.

Factor analysis identified four factors used to determine the main characteristics of the rehabilitation of ATO participants and made up 80.37 % of the total variance (Table 3). As shown in Table 3, the composition of the first component (37.9 %) included the most ingredients: 1) Development of a physical and mental health rehabilitation program; 2) Availability of sanatorium-resort treatment; 3) Accessibility to discounted purchase of medicines; 4) Provision of a psychologist at the place of service; 5) Provision of highly specialized assistance to ATO veterans; 6) Referral for treatment at the Medical Center; and 7) Free medical care. The second and fourth components accounted for 11.89 % and 9.64 % of the sample variance, respectively: 1) Allocation of funds for program implementation; 2) Creation of material and technical conditions for assisting ATO veterans; and 3) Provision of highly specialized assistance to ATO veterans (Table 3). The third component accounted for 9.64 % of the sample’s variance, and included 1) Priority and availability of medical care for ATO veterans; 2) Accessibility to discounted purchase of medicines; 3) Independent application for medical assistance; 4) Comfortable conditions for ATO veterans; and 5) Compliance with the doctor’s recommendations (Table 3).

**Table 3:** Matrix of constituent main characteristics of rehabilitation of ATO veterans.

Item number	Answers received from health care organizers regarding the benefits of the rehabilitation system for ATO veterans	Components			
		1	2	3	4
1	Allocation of funds for the implementation of the program	0.096	**0.731**	−0.249	**0.624**
2	Priority and availability of medical care for ATO participants	0.344	−0.314	**0.641**	0.460
3	Development of a physical and mental health rehabilitation program	**0.886**	−0.214	0.541	0.460
4	Creation of material and technical conditions for providing assistance to ATO veterans	0.412	**0.600**	−0.147	**0.668**
5	Availability of sanatorium-resort treatment	**0.825**	−0.420	−0.367	0.055
6	Availability of discounted drugs	**0.659**	0.308	**0.601**	0.258
7	Availability of a psychologist at the place of service	**0.709**	0.455	−0.147	0.104
8	Providing highly specialized care to ATO veterans	**0.616**	**0.685**	−0.142	−0.336
9	Referral for treatment at the Medical Rehabilitation Center	**0.970**	−0.149	0.158	−0.104
10	Free medical care	**0.729**	0.281	−0.598	−0.157
11	Comfortable conditions for ATO veterans	0.403	0.337	**0.798**	−0.036
12	Highly qualified medical specialists	0.591	0.516	0.376	−0.477
13	Independent application for medical assistance	0.475	−0.466	**0.701**	0.040
14	Following the doctor’s recommendations	0.409	0.328	**0.657**	0.514

Abbreviations: ATO: anti-terrorist operation.

The correlation matrix of the advantages of the rehabilitation system is shown in Table 4. Correlations were found between ‘Provision of ATO veterans with legally defined guarantees’ and ‘Provision of ATO veterans with legally defined guarantees: medical (r = 0.916) and social (r = 0.582) protection’, ‘Provision of comprehensive medical rehabilitation’ (r = 0.607), ‘Provision of complex psychological rehabilitation’ (r = 0.554), as well as ‘Creation of a program of medical assistance’ (r = 0.580). ‘Provision of ATO veterans with legally defined guarantees (medical protection)’ was correlated with ‘Effective functioning of regional centers of rehabilitation assistance’ (r = 0.607) and ‘Creation of a program for the provision of medical assistance’ (r = 0.542). Three questions related to the provision of comprehensive rehabilitation: ‘Provision of complex psychological rehabilitation’ (r = 0.758), ‘Provision of comprehensive physical rehabilitation’ (r = 0.702), and ‘Implementation of a multidisciplinary approach’ (r = 0.702). Additionally, a multidisciplinary approach to rehabilitation correlates with the creation of a medical care program (r = 0.678), experience working with combat trauma (r = 0.670), and psychological rehabilitation (r = 0.736). ‘Experience of working with combat trauma’ had a positive relationship with ‘Performance of comprehensive psychological rehabilitation’ (r = 0.690). ‘Establishment of a medical assistance program’ correlates with ‘Ensuring comprehensive physical rehabilitation’ (r = 0.678).

**Table 4:** Interrelationships of the benefits of the rehabilitation system for ATO veterans.

Item number	Advantages of the rehabilitation system for ATO veterans	1	2	3	4	5	6	7	8	9	10	11
**1**	Providing veterans of the anti-terrorist operation with legally defined guarantees	1.000										
**2**	Providing veterans of the anti-terrorist operation with legally defined guarantees (medical protection)	**0.916**	1.000									
**3**	Provision of statutory guarantees for ATO veterans (social protection)	**0.582**	0.494	1.000								
**4**	Effective functioning of regional centers of rehabilitation assistance	0.468	**0.607**	0.032	1.000							
**5**	Provision of comprehensive medical rehabilitation	**0.607**	0.420	0.445	0.464	1.000						
**6**	Provision of complex psychological rehabilitation	**0.554**	0.374	0.028	0.175	**0.758**	1.000					
**7**	Provision of comprehensive physical rehabilitation	0.468	0.250	0.352	0.033	**0.702**	0.600	1.000				
**8**	Creation of a medical assistance program	**0.580**	**0.542**	0.069	0.145	0.580	0.592	**0.678**	1.000			
**9**	Implementation of a multidisciplinary approach	0.468	0.429	0.192	0.200	**0.702**	**0.736**	0.375	**0.678**	1.000		
**10**	Experience of rehabilitation work in case of combat injury	−0.282	−0.287	−0.037	−0.230	−0.486	**0.690**	−0.192	−0.260	**0.670**	1.000	
**11**	Perception of ATO veterans as heroes of our time	−0.038	−0.020	0.311	0.038	0.229	0.062	0.250	−0.077	0.071	−0.561	1.000

Abbreviations: ATO: anti-terrorist operation.

Based on the results of the factor analysis, we identified four main factors F1-F4 (Table 5), contributing 79.50 % of the total variance. The first component, accounting for 42.80 % of the total variance, included the following items: 1) Provision of ATO veterans with legally defined guarantees; 2) Provision of ATO veterans with legally defined guarantees (medical protection); 3) Provision of complex rehabilitation measures in the medical, physical and psychological spheres; 4) Creation of a medical assistance program; 5) Implementation of a multidisciplinary approach to rehabilitation supported by legal guarantees; and 6) Experience in rehabilitation work in the event of a combat injury. The second component, accounting for 14.20 % of the sample variance, included: 1) Provision of ATO veterans with legally defined guarantees (social protection); 2) Effective functioning of regional centers; 3) Experience in rehabilitation work in the event of a combat injury; and 4) Perception of ATO veterans as heroes of our time. The third component, which accounts for 13.34 % of the total variance, consists of two elements with a positive value: 1) Perception of ATO veterans as heroes of our time; and 2) Provision of ATO veterans with legally defined guarantees (social protection). The fourth component, accounting for 9.15 % of the total sample variance, included four items: 1) Provision of complex medical rehabilitation; 2) Provision of complex psychological rehabilitation; 3) Provision of complex physical rehabilitation; and 4) Creation of a medical care program.

**Table 5:** Matrix of component advantages of the rehabilitation system of ATO veterans.

Item number	The characteristics of the rehabilitation system are defined	Components			
		1	2	3	4
1	Providing veterans of the anti-terrorist operation with legally defined guarantees	**0.817**	0.486	−0.125	−0.055
2	Providing veterans of the anti-terrorist operation with legally defined guarantees (medical protection)	**0.740**	0.489	−0.085	−0.314
3	Provision of statutory guarantees for ATO veterans (social protection)	0.430	**0.634**	**0.607**	0.325
4	Effective functioning of regional centers of rehabilitation assistance	0.487	**0.597**	0.204	−0.306
5	Provision of comprehensive medical rehabilitation	**0.744**	−0.300	0.034	**0.604**
6	Provision of complex psychological rehabilitation	**0.865**	−0.030	0.055	**0.662**
7	Provision of comprehensive physical rehabilitation	**0.813**	−0.375	−0.167	**0.734**
8	Creation of a medical assistance program	**0.636**	−0.082	−0.716	**0.726**
9	Implementation of a multidisciplinary approach	**0.745**	−0.115	−0.480	0.165
10	Experience of rehabilitation work in case of combat injury	**0.809**	**0.616**	−0.115	−0.129
11	Perception of ATO veterans as heroes of our time	−0.348	**0.790**	**0.675**	0.500

Abbreviations: ATO: anti-terrorist operation.

The correlation matrix of disadvantages is shown in Table 6. ‘Violation of legislation regulating the provision of medical care to war veterans’ was correlated with ‘Insufficient integrated approach in the treatment and rehabilitation of veterans’ (r = 0.658) and ‘Insufficient financing of health care facilities’ (r = 0.588). ‘Loss of the social status of a war veteran at the primary level of medical care’ was correlated with ‘Loss of dispensary supervision of veterans’ health’ (r = 0.531) and ‘Lack of a guaranteed rehabilitation package for ATO veterans’ (r = 0.537). ‘Insufficient integrated approach in the treatment and rehabilitation of veterans’ correlates with ‘Insufficient financing of health care facilities’ (r = 0.690) and ‘Insufficient opportunity to update the material and technical base’ (r = 0.597). ‘Insufficient financing of health care facilities’ is associated with ‘Insufficient opportunity to update the material and technical base’ (r = 0.654). ‘Lack of a guaranteed rehabilitation package for ATO veterans’ is related to ‘Low salaries of specialists’ (r = 0.794).

**Table 6:** Interrelationships of disadvantages of the system of rehabilitation of ATO veterans.

Item number	Disadvantages of the rehabilitation system for ATO veterans	1	2	3	4	5	6	7	8
1	Violation of legislation regulating the provision of medical care to war veterans	1.000							
2	Loss of social status of war veterans at primary health care level	0.309	1.000						
3	Loss of dispensary supervision of veterans’ health	0.079	**0.531**	1.000					
4	An insufficient comprehensive approach in the treatment and rehabilitation of ATO veterans	**0.658**	−0.26	−0.088	1.000				
5	Insufficient financing of health care facilities	**0.588**	−0.110	−0.385	**0.690**	1.000			
6	Lack of a guaranteed rehabilitation package for ATO veterans	−0.197	**0.537**	0.385	0.045	−0.023	1.000		
7	Insufficient opportunity to update the material and technical base	0.172	0.000	0.107	**0.597**	**0.654**	0.334	1.000	
8	Low salaries of specialists	−0.273	0.394	0.190	0.060	−0.022	**0.794**	0.106	1.000

Abbreviations: ATO: anti-terrorist operation.

Further factor analysis made it possible to identify three main components, and key links in the shortcomings of the ATO veterans’ rehabilitation system that account for 92.65 % of the total variance (Table 7). The first factor explained 9.15 % of the total sample variance, which includes: 1) Loss of dispensary supervision of the health status of ATO veterans; and 2) Insufficient comprehensive approach in the treatment and rehabilitation of ATO veterans. The second component was loaded with the following components: 1) Insufficient financing of healthcare facilities; 2) Lack of a guaranteed rehabilitation package for ATO veterans; 3) Insufficient opportunity to update the material and technical base; and 4) Low wages of specialists. The third component was characterized by: 1) Violation of legislation regulating the provision of medical assistance to war veterans; and 2) Loss of the social status of a war veteran at the primary level of medical care.

**Table 7:** The matrix of component shortcomings of the rehabilitation of ATO veterans.

Item number	The characteristics of the rehabilitation system are defined	Components		
		1	2	3
1	Violation of legislation regulating the provision of medical care to war veterans	0.443	−0.028	**0.709**
2	Loss of social status of a war veteran at the primary level of medical care	−0.616	0.116	**0.669**
3	Loss of dispensary supervision of veterans’ health	**0.849**	0.462	−0.006
4	An insufficient comprehensive approach in the treatment and rehabilitation of ATO veterans	**0.907**	0.353	−0.045
5	Insufficient financing of health care facilities	−0.633	**0.706**	−0.096
6	Lack of a guaranteed rehabilitation package for ATO veterans	0.231	**0.780**	0.394
7	Insufficient opportunity to update the material and technical base	0.463	**0.813**	0.124
8	Low salaries of specialists	−0.451	**0.681**	−0.457

Abbreviations: ATO: anti-terrorist operation.

The correlations between factors were below 0.7, confirming discriminant validity. Internal consistency was assessed using Cronbach’s alpha, with an α value exceeding 0.75, indicating acceptable reliability.

## 4 Discussion

Factor analysis enabled us to evaluate the satisfaction level of ATO veterans in Ukraine regarding the rehabilitation system using our proposed blocks. These blocks, including the key features, benefits, and drawbacks of the rehabilitation system, allowed us to identify and structure the main components of the rehabilitation process, which can aid in optimizing the current system in Ukraine.

The first component of the main characteristics, named ‘Accessibility’, included good access to rehabilitation assistance independent from geographic, economic, social, and organizational barriers. The second component, ‘Financing the Rehabilitation Programs for ATO Participants’, included the allocation of funds, the establishment of necessary resources for assistance, and the provision of specialized care for ATO veterans. The third component included: the availability of discounted drugs; priority and availability of medical care for ATO participants; independent application for medical assistance; comfortable conditions for ATO veterans; following the doctor’s recommendations, and was defined as ‘Commitment of ATO veterans to rehabilitation’.

The second block focused on the benefits of the rehabilitation system. The first component was named ‘Regulatory and legal support of medical, social protection of the rehabilitation of veterans’. This component underscores the importance of legal frameworks and social protections in facilitating veterans’ access to rehabilitation services. The second component consists of providing veterans of the anti-terrorist operation with legally defined guarantees, and effective functioning of rehabilitation assistance, so we called this component ‘Experienced personnel who carry out rehabilitation for veterans’. This emphasizes the significance of skilled professionals in delivering rehabilitation services tailored to veterans’ needs. The third component was named ‘Respectful attitude to veterans’ and the fourth component combined elements related to the care provision and was called ‘Comprehensive rehabilitation’.

The doctors deemed the block of deficiencies in the organization of rehabilitation measures significant. Subsequently, factor analysis was employed to isolate the three primary components that constituted the major shortcomings in rehabilitating ATO veterans. The first factor was characterized by the insufficient comprehensive approach to the treatment and rehabilitation of ATO veterans, and the absence of dispensary supervision of their health, which constituted the most significant burden. An inadequate comprehensive approach reflects a disregard and infringement on a patient-centered approach and the uninterrupted delivery of medical care. Therefore, this factor can be labeled as a ‘Violation of the integral principle’. The second component was insufficient financing of health care, which was called ‘Insufficient material and technical support and financing of rehabilitation’. The third component, ‘Gaps in legislative and regulatory provision of rehabilitation of ATO participants’, included the violations of the legislation regulating the provision of medical care to war veterans and the loss of the social status of a war veteran at the primary level of medical care.

As shown in Fig. 1, all blocks are unequally connected, creating a complete scheme of the existing rehabilitation system, allowing for a comprehensive assessment. A consequence of commitment to rehabilitation is that ATO veterans seek medical care on their own, which contributes to a more effective recovery process.

The role of accessibility in veteran rehabilitation is noted by Elaine J. Mahoney et al. [20] in their study of rehabilitation needs five years after traumatic brain injury, and by Aniz Agha [21] in her study of care for older veterans. Accessibility is closely related to the allocation of money for rehabilitation programs on its material and technical side since finances cover economic affordability [22].

Different empirical studies show that the degree of engagement in clashes is closely related to the outcome of combat-related guilt [23,24]. Feelings of guilt, in turn, can accelerate the development of symptoms of post-traumatic stress disorder [25]. This often leads to avoidance behaviors (e.g., social isolation, substance abuse) to avoid guilt. Not only does chronic guilt tend to increase intrusion, distress, and avoidant coping - the three hallmarks of PTSD - it also appears to interfere with help-seeking and response to treatment [26,27].

Researchers who have investigated the consequences of hostilities resulting in a high incidence of disability related to limb amputation have emphasized the importance of the holistic approach in providing rehabilitation services. The U.S. Army’s amputee care programs, which have specialized centers staffed with multidisciplinary care teams, have redefined the standard for rehabilitating limb loss, recognizing that limb reconstruction is only one part of a patient’s complete recovery and reintegration [28]. Drawing inspiration from this approach, comprehensive rehabilitation programs have achieved success in rehabilitating patients with severe limb injuries requiring limb reconstruction [28]. For example, in Ukraine, rehabilitation centers are being created in sanatoriums, and departments are being opened in hospitals. As a holistic option for veteran rehabilitation, Hope In Health’s Veterans Recovery Program has been specifically designed to support veterans’ unique mental and physical recovery needs and their ecosystem of loved ones and mates. Unique aspects of the Ukrainian approach to veteran rehabilitation issues lie in the country’s health care system. During the military actions of Russia in Ukraine, the rehabilitation system has undergone changes as veterans arrive from the battlefield. The factor analysis we conducted serves as a form of monitoring and evaluation of the rehabilitation system from the perspective of the doctors directly involved in it. While resource-intensive and not universally available in healthcare systems, civilian trauma practitioners can learn from this approach to prioritize patient recovery [28].

This study has a limited sample size, which may restrict the generalizability of the results. Specifically, the small sample size may limit the ability to capture a wider range of perspectives. Further, with a sample concentrated in a specific geographic region, our findings may not fully reflect the experiences and practices of doctor-organizers. Thus, caution should be exercised when applying the findings to other settings. However, the ATO in the east of Ukraine is a complex of military and special organizational and legal measures of the Ukrainian forces, aimed at countering the activities of illegal Russian and pro-Russian armed groups in the war in the east of Ukraine. The current study identified and structured the main components of the rehabilitation process, which can aid in optimizing the current rehabilitation system in Ukraine.

## 5 Conclusions

The key features of ATO veteran rehabilitation include accessibility, funding for rehabilitation programs, and dedication to the rehabilitation of veterans. The advantages of such rehabilitation programs include regulatory and legal provisions for the medical and social protection of veterans, experienced personnel, respectful attitudes towards veterans, and comprehensive rehabilitation. However, the rehabilitation system also has significant drawbacks, such as inadequate material and technical support, insufficient funding for rehabilitation, and gaps in legislative and regulatory provisions for ATO veteran rehabilitation measures. Therefore, increasing funding and improving support for rehabilitation programs, as well as addressing the gaps in legislative and regulatory provisions, may help improve the effectiveness of ATO veteran rehabilitation programs. Specifically, attracting the attention of political figures and local self-government bodies to legislative documents aimed at addressing the physical, medical, psychological, and social health needs of veterans is needed. These findings can aid in optimizing the current rehabilitation system in Ukraine.

## CRediT authorship contribution statement

**Irina Holovanova:** Writing – review & editing, Writing – original draft, Supervision, Project administration, Methodology, Formal analysis, Data curation, Conceptualization. **Oleksandr Havlovsky:** Writing – review & editing, Writing – original draft, Methodology, Formal analysis, Data curation, Conceptualization. **Shanshan Wang:** Writing – review & editing, Writing – original draft. **Oleksandr Korneta:** Writing – review & editing, Data curation, Conceptualization. **Maksym Khorosh:** Writing – review & editing, Data curation, Conceptualization. **Igor Kaydashev:** Writing – review & editing, Data curation, Conceptualization. **Renee Robinson:** Writing – review & editing, Writing – original draft. **Ubydul Haque:** Writing – review & editing, Writing – original draft, Supervision, Project administration, Data curation, Conceptualization.

## Ethics approval and consent to participate

Ethical approval was obtained from Poltava State Medical University, Ukraine (ethics approval number: No. 292). Informed consent was obtained from each participant.

## Availability of data and materials

A data set is available based on request.

## Funding

None.

## Declaration of competing interest

The authors declare that they have no known competing financial interests or personal relationships that could have appeared to influence the work reported in this paper.
