# Supplementary material for: Doctors’ satisfaction with the rehabilitation system for anti-terrorist operation participants: A factor analysis
Source: Heliyon. 2024 Nov 23;11(1):e40667. doi: 10.1016/j.heliyon.2024.e40667 (PMC11721236; doi:10.1016/j.heliyon.2024.e40667)
Supplement: Multimedia component 1 [file mmc1.pdf]

**Average points received from respondents regarding the main characteristics of the rehabilitation system for ATO veterans**

| ***Questions for health care organizers regarding the*** main characteristics ***of the rehabilitation system for ATO veterans*** | ***Points (mean Max-min )*** |
|---|---|
| Allocation of funds for the implementation of the program | 4.3 (3-5) |
| Priority and availability of medical care for ATO participants | 3.7 (2-5) |
| Development of a physical and mental health rehabilitation program | 3.8 (2-5) |
| Creation of material and technical conditions for providing assistance to ATO veterans | 4.2 (3-5) |
| Availability of sanatorium-resort treatment | 3.8 (2-5) |
| Availability of discounted drugs | 3.6 (0-5) |
| Availability of a psychologist at the place of service | 3.6 (0-5) |
| Providing highly specialized care to ATO veterans | 4.3 (2-5) |
| Referral for treatment at the Medical Rehabilitation Center | 3.8 (3-5) |
| Free medical care | 4.5 (3.5) |
| Comfortable conditions for ATO veterans | 4.4 (3-5) |
| Highly qualified medical specialists | 4.3 (3-5) |
| Independent application for medical assistance | 3.8 (3-5) |
| Following the doctor's doctor's recommendations | 4.2 (2-5) |

**Average points received from respondents regarding the *benefits* characteristics of the rehabilitation system for ATO veterans**

| ***Questions for health care organizers regarding the benefits of the rehabilitation system for ATO veterans*** | ***Points (mean Max-min )*** |
|---|---|
| Providing veterans of the anti-terrorist operation with legally defined guarantees | 3.8 (3-5) |
| Providing veterans of the anti-terrorist operation with legally defined guarantees (medical protection) | 3.7 (3-5) |
| Provision of statutory guarantees for ATO veterans (social protection) | 3.6 (3-5) |
| Effective functioning of regional centers of rehabilitation assistance | 3.9 (3-5) |
| Provision of comprehensive medical rehabilitation | 3.2 (2-5) |
| Provision of complex psychological rehabilitation | 3.3 (2-5) |
| Provision of comprehensive physical rehabilitation | 3.3 (2-5) |
| Creation of a medical assistance program | 3.3 (2-5) |
| Implementation of a multidisciplinary approach | 3.2 (2-5) |
| Experience of rehabilitation work in case of combat injury | 2.7 (1-5) |
| Perception of ATO veterans as heroes of our time | 3.6 (2-5) |

**Average points received from respondents regarding the** disadvantages **characteristics of the rehabilitation system for ATO veterans**

| ***Questions for health care organizers regarding the*** disadvantages ***of the rehabilitation system for ATO veterans*** | ***Points (mean Max-min )*** |
|---|---|
| Violation of legislation regulating the provision of medical care to war veterans | 3.2 (2-5) |
| Loss of social status of war veterans at primary health care level | 3.1 (1-5) |
| Loss of dispensary supervision of veterans' health | 2.8 (1-4) |
| An insufficient comprehensive approach in the treatment and rehabilitation of ATO veterans | 2.7 (1-3) |
| Insufficient financing of health care facilities | 2.6 (1-5) |
| Lack of a guaranteed rehabilitation package for ATO veterans | 4.5 (2-5) |
| Insufficient opportunity to update the material and technical base | 2.5 (1-4) |
| Low salaries of specialists | 3.0 (1-5) |
